# 2,4-Dihydr­oxy-*N*′-(4-methoxy­benzyl­idene)benzohydrazide

**DOI:** 10.1107/S1600536808001104

**Published:** 2008-01-18

**Authors:** Yun-Peng Diao, Shan-Shan Huang, Jian-Kui Zhang, Ting-Guo Kang

**Affiliations:** a School of Pharmacy, Dalian Medical University, Dalian 116044, People’s Republic of China; b Liaoning University of Traditional Chinese Medicine, Shenyang 110032, People’s Republic of China

## Abstract

The mol­ecule of the title compound, C_15_H_14_N_2_O_4_, displays a *trans* configuration with respect to the hydrazide C=N bond. The dihedral angle between the two benzene rings is 15.0 (2)°. In the crystal structure, mol­ecules are linked through inter­molecular O—H⋯N and O—H⋯O hydrogen bonds, forming layers parallel to the *ab* plane; an intramolecular N—H⋯O hydrogen bond is also present.

## Related literature

For the biological properties of Schiff base compounds, see: Brückner *et al.* (2000 ▶); Harrop *et al.* (2003 ▶); Ren *et al.* (2002 ▶). For related structures, see: Diao (2007 ▶); Diao *et al.* (2007 ▶); Li *et al.* (2007 ▶); Huang *et al.* (2007 ▶).
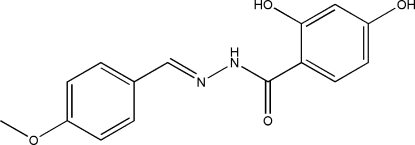


## Experimental

### 

#### Crystal data

C_15_H_14_N_2_O_4_
*M* *_r_* = 286.28
Orthorhombic, 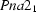

*a* = 12.494 (3) Å
*b* = 5.196 (1) Å
*c* = 20.825 (4) Å
*V* = 1351.9 (5) Å^3^
*Z* = 4
Mo *K*α radiation
μ = 0.10 mm^−1^
*T* = 298 (2) K
0.22 × 0.20 × 0.20 mm

#### Data collection

Bruker SMART CCD area-detector diffractometer
Absorption correction: multi-scan (*SADABS*; Bruker, 2000 ▶) *T*_min_ = 0.978, *T*_max_ = 0.980
7661 measured reflections
1519 independent reflections
1382 reflections with *I* > 2σ(*I*)
*R*_int_ = 0.025

#### Refinement

*R*[*F* ^2^ > 2σ(*F* ^2^)] = 0.031
*wR*(*F* ^2^) = 0.079
*S* = 1.06
1519 reflections
196 parameters
2 restraints
H atoms treated by a mixture of independent and constrained refinement
Δρ_max_ = 0.13 e Å^−3^
Δρ_min_ = −0.15 e Å^−3^

### 

Data collection: *SMART* (Bruker, 2000 ▶); cell refinement: *SAINT* (Bruker, 2000 ▶); data reduction: *SAINT*; program(s) used to solve structure: *SHELXTL* (Sheldrick, 2008 ▶); program(s) used to refine structure: *SHELXTL*; molecular graphics: *SHELXTL*; software used to prepare material for publication: *SHELXTL*.

## Supplementary Material

Crystal structure: contains datablocks global, I. DOI: 10.1107/S1600536808001104/rz2192sup1.cif

Structure factors: contains datablocks I. DOI: 10.1107/S1600536808001104/rz2192Isup2.hkl

Additional supplementary materials:  crystallographic information; 3D view; checkCIF report

## Figures and Tables

**Table 1:** Hydrogen-bond geometry (Å, °)

*D*—H⋯*A*	*D*—H	H⋯*A*	*D*⋯*A*	*D*—H⋯*A*
N2—H2*A*⋯O3	0.901 (10)	1.94 (2)	2.646 (2)	134 (3)
O4—H4⋯O2^i^	0.82	1.85	2.671 (2)	174
O3—H3⋯N1^ii^	0.82	2.48	3.234 (2)	154
O3—H3⋯O2^ii^	0.82	2.14	2.788 (2)	136

Symmetry codes: (i) 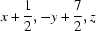; (ii) 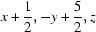.

**Fig. 1.**
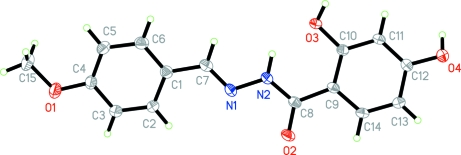
The structure of the title compound with displacement ellipsoids drawn at the 30% probability level.

**Fig. 2.**
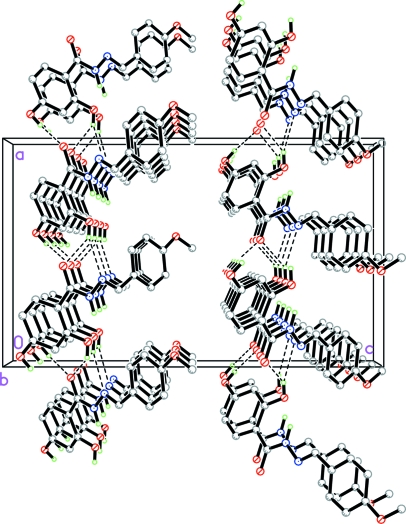
Crystal packing of the title compound viewed along the b axis. Intermolecular hydrogen bonds are shown as dashed lines.

## supplementary crystallographic information

### Comment

Schiff base compounds have received much attention in recent years. Some Schiff base metal complexes have been found to have pharmacological and antitumor properties (Brückner *et al.*, 2000; Harrop *et al.*, 2003; Ren *et al.*, 2002). As part of our research programme on the synthesis and characterization of Schiff base compounds (Diao *et al.*, 2007; Diao, 2007; Li *et al.*, 2007; Huang *et al.*, 2007), we report here the structure of the title ligand.

The molecule of the title compound displays a *trans* configuration with respect to the C=N bond (Fig. 1). The dihedral angle between the two benzene rings is 15.0 (2)°. The molecular conformation is stabilized by an intramolecular N—H···O hydrogen interaction (Table 1). In the crystal, molecules are linked through intermolecular O—H···N and O—H···O hydrogen bonds (Table 1), forming layers parallel to the *ab* plane (Fig. 2).

### Experimental

4-Methoxybenzaldehyde (0.1 mmol, 13.6 mg) and 2,4-dihydroxybenzoic acid hydrazide (0.1 mmol, 16.8 mg) were dissolved in a 95% ethanol solution (10 ml). The mixture was stirred at room temperature to give a clear colourless solution. Crystals of the title compound were formed by gradual evaporation of the solvent over a period of three days at room temperature.

### Refinement

Atom H2A was located in a difference Fourier map and refined isotropically, with the N—H distance restrained to 0.90 (1) Å. All other H atoms were placed in idealized positions and constrained to ride on their parent atoms, with O—H = 0.82 Å, C—H = 0.93–0.96 Å, and with *U*_iso_(H) = 1.2*U*_eq_(C) or 1.5*U*_eq_(C, O) for methyl and hydroxy H atoms. In the absence of significant anomalous scattering effects, Friedel opposites were merged in the final refinement.

### Crystal data

**Table:**

C_15_H_14_N_2_O_4_	*F*_000_ = 600
*M**_r_* = 286.28	*D*_x_ = 1.407 Mg m^−^^3^
Orthorhombic, *P**n**a*2_1_	Mo *K*α radiation λ = 0.71073 Å
Hall symbol: P 2c -2n	Cell parameters from 3064 reflections
*a* = 12.494 (3) Å	θ = 2.7–27.2º
*b* = 5.196 (1) Å	µ = 0.10 mm^−^^1^
*c* = 20.825 (4) Å	*T* = 298 (2) K
*V* = 1351.9 (5) Å^3^	Block, colourless
*Z* = 4	0.22 × 0.20 × 0.20 mm

### Data collection

**Table:**

Bruker SMART CCD area-detector diffractometer	1519 independent reflections
Radiation source: fine-focus sealed tube	1382 reflections with *I* > 2σ(*I*)
Monochromator: graphite	*R*_int_ = 0.025
*T* = 298(2) K	θ_max_ = 27.0º
ω scans	θ_min_ = 2.0º
Absorption correction: multi-scan(SADABS; Bruker, 2000)	*h* = −15→15
*T*_min_ = 0.978, *T*_max_ = 0.980	*k* = −6→5
7661 measured reflections	*l* = −25→26

### Refinement

**Table:**

Refinement on *F*^2^	Secondary atom site location: difference Fourier map
Least-squares matrix: full	Hydrogen site location: inferred from neighbouring sites
*R*[*F*^2^ > 2σ(*F*^2^)] = 0.031	H atoms treated by a mixture of independent and constrained refinement
*wR*(*F*^2^) = 0.079	*w* = 1/[σ^2^(*F*_o_^2^) + (0.0468*P*)^2^ + 0.123*P*] where *P* = (*F*_o_^2^ + 2*F*_c_^2^)/3
*S* = 1.06	(Δ/σ)_max_ < 0.001
1519 reflections	Δρ_max_ = 0.13 e Å^−^^3^
196 parameters	Δρ_min_ = −0.15 e Å^−^^3^
2 restraints	Extinction correction: none
Primary atom site location: structure-invariant direct methods	

### Special details

**Table:**

Geometry. All e.s.d.'s (except the e.s.d. in the dihedral angle between two l.s. planes) are estimated using the full covariance matrix. The cell e.s.d.'s are taken into account individually in the estimation of e.s.d.'s in distances, angles and torsion angles; correlations between e.s.d.'s in cell parameters are only used when they are defined by crystal symmetry. An approximate (isotropic) treatment of cell e.s.d.'s is used for estimating e.s.d.'s involving l.s. planes.
Refinement. Refinement of *F*^2^ against ALL reflections. The weighted *R*-factor *wR* and goodness of fit *S* are based on *F*^2^, conventional *R*-factors *R* are based on *F*, with *F* set to zero for negative *F*^2^. The threshold expression of *F*^2^ > σ(*F*^2^) is used only for calculating *R*-factors(gt) *etc*. and is not relevant to the choice of reflections for refinement. *R*-factors based on *F*^2^ are statistically about twice as large as those based on *F*, and *R*- factors based on ALL data will be even larger.

### Fractional atomic coordinates and isotropic or equivalent isotropic displacement parameters (Å^2^)

**Table:**

	*x*	*y*	*z*	*U*_iso_*/*U*_eq_	
O1	−0.07270 (13)	0.0953 (3)	0.95352 (10)	0.0550 (5)	
O2	0.05579 (11)	1.2801 (3)	0.67387 (9)	0.0461 (4)	
O3	0.37031 (11)	1.3774 (3)	0.73708 (8)	0.0411 (4)	
H3	0.4359	1.3777	0.7357	0.062*	
O4	0.41377 (12)	2.0606 (3)	0.58664 (8)	0.0438 (4)	
H4	0.4607	2.1038	0.6120	0.066*	
N1	0.11530 (13)	0.9630 (3)	0.76750 (9)	0.0358 (4)	
N2	0.18526 (13)	1.1298 (3)	0.73763 (9)	0.0354 (4)	
C1	0.09837 (17)	0.6230 (4)	0.84516 (11)	0.0361 (4)	
C2	−0.01216 (19)	0.5952 (5)	0.83938 (12)	0.0461 (6)	
H2	−0.0499	0.6974	0.8105	0.055*	
C3	−0.06553 (18)	0.4174 (5)	0.87614 (13)	0.0490 (6)	
H3A	−0.1392	0.3994	0.8717	0.059*	
C4	−0.01114 (18)	0.2637 (4)	0.91993 (11)	0.0394 (5)	
C5	0.09840 (18)	0.2886 (4)	0.92608 (12)	0.0421 (5)	
H5	0.1360	0.1855	0.9548	0.051*	
C6	0.15162 (17)	0.4694 (4)	0.88882 (12)	0.0424 (5)	
H6	0.2253	0.4876	0.8934	0.051*	
C7	0.15821 (17)	0.8097 (4)	0.80745 (11)	0.0377 (5)	
H7	0.2320	0.8173	0.8129	0.045*	
C8	0.15001 (14)	1.2894 (4)	0.69171 (10)	0.0317 (4)	
C9	0.22691 (14)	1.4776 (4)	0.66456 (10)	0.0304 (4)	
C10	0.33078 (14)	1.5226 (4)	0.68779 (10)	0.0306 (4)	
C11	0.39332 (15)	1.7176 (4)	0.66248 (10)	0.0324 (4)	
H11	0.4610	1.7489	0.6793	0.039*	
C12	0.35562 (16)	1.8657 (4)	0.61231 (10)	0.0335 (4)	
C13	0.25493 (16)	1.8182 (4)	0.58614 (11)	0.0394 (5)	
H13	0.2303	1.9129	0.5512	0.047*	
C14	0.19282 (16)	1.6282 (4)	0.61303 (10)	0.0373 (5)	
H14	0.1250	1.5987	0.5961	0.045*	
C15	−0.0203 (2)	−0.0748 (5)	0.99722 (14)	0.0540 (6)	
H15A	0.0135	0.0235	1.0306	0.081*	
H15B	0.0328	−0.1729	0.9746	0.081*	
H15C	−0.0720	−0.1893	1.0159	0.081*	
H2A	0.2547 (11)	1.133 (6)	0.7492 (17)	0.080*	

### Atomic displacement parameters (Å^2^)

**Table:**

	*U*^11^	*U*^22^	*U*^33^	*U*^12^	*U*^13^	*U*^23^
O1	0.0502 (9)	0.0531 (9)	0.0618 (11)	−0.0010 (8)	0.0024 (9)	0.0263 (8)
O2	0.0247 (7)	0.0480 (8)	0.0657 (11)	−0.0043 (6)	−0.0063 (7)	0.0119 (8)
O3	0.0221 (6)	0.0521 (9)	0.0490 (9)	−0.0013 (6)	−0.0039 (6)	0.0161 (8)
O4	0.0398 (8)	0.0458 (9)	0.0458 (8)	−0.0114 (7)	−0.0038 (7)	0.0110 (7)
N1	0.0311 (8)	0.0368 (9)	0.0395 (9)	−0.0081 (7)	0.0034 (7)	0.0004 (8)
N2	0.0258 (8)	0.0401 (9)	0.0404 (9)	−0.0046 (7)	0.0007 (8)	0.0049 (7)
C1	0.0364 (10)	0.0354 (10)	0.0364 (10)	−0.0019 (8)	−0.0005 (8)	−0.0012 (8)
C2	0.0383 (11)	0.0517 (13)	0.0481 (13)	−0.0061 (10)	−0.0100 (10)	0.0175 (11)
C3	0.0346 (10)	0.0557 (13)	0.0567 (14)	−0.0070 (10)	−0.0084 (11)	0.0186 (11)
C4	0.0422 (12)	0.0360 (10)	0.0399 (11)	−0.0009 (9)	0.0012 (9)	0.0062 (9)
C5	0.0439 (12)	0.0400 (11)	0.0425 (11)	0.0066 (9)	−0.0042 (9)	0.0068 (10)
C6	0.0347 (10)	0.0451 (12)	0.0476 (13)	0.0029 (9)	−0.0033 (10)	0.0006 (10)
C7	0.0311 (10)	0.0418 (11)	0.0404 (11)	−0.0033 (9)	−0.0001 (9)	−0.0023 (9)
C8	0.0239 (9)	0.0318 (9)	0.0394 (10)	0.0013 (7)	0.0020 (8)	−0.0037 (8)
C9	0.0233 (8)	0.0324 (9)	0.0356 (10)	0.0009 (7)	0.0000 (8)	−0.0020 (8)
C10	0.0240 (8)	0.0333 (9)	0.0346 (10)	0.0032 (7)	0.0006 (8)	0.0007 (8)
C11	0.0227 (8)	0.0371 (10)	0.0376 (10)	−0.0001 (7)	−0.0016 (8)	−0.0006 (8)
C12	0.0296 (9)	0.0346 (10)	0.0363 (10)	−0.0020 (8)	0.0024 (8)	−0.0004 (8)
C13	0.0333 (10)	0.0460 (11)	0.0389 (11)	−0.0003 (9)	−0.0065 (9)	0.0089 (10)
C14	0.0262 (9)	0.0437 (11)	0.0419 (11)	−0.0031 (8)	−0.0081 (8)	0.0019 (9)
C15	0.0663 (16)	0.0453 (12)	0.0504 (14)	0.0093 (13)	0.0052 (13)	0.0159 (11)

### Geometric parameters (Å, °)

**Table:**

O1—C4	1.359 (3)	C4—C5	1.381 (3)
O1—C15	1.427 (3)	C5—C6	1.388 (3)
O2—C8	1.235 (2)	C5—H5	0.9300
O3—C10	1.366 (2)	C6—H6	0.9300
O3—H3	0.8200	C7—H7	0.9300
O4—C12	1.356 (2)	C8—C9	1.483 (3)
O4—H4	0.8200	C9—C14	1.394 (3)
N1—C7	1.270 (3)	C9—C10	1.405 (3)
N1—N2	1.379 (2)	C10—C11	1.384 (3)
N2—C8	1.340 (3)	C11—C12	1.380 (3)
N2—H2A	0.901 (10)	C11—H11	0.9300
C1—C6	1.381 (3)	C12—C13	1.393 (3)
C1—C2	1.394 (3)	C13—C14	1.375 (3)
C1—C7	1.455 (3)	C13—H13	0.9300
C2—C3	1.373 (3)	C14—H14	0.9300
C2—H2	0.9300	C15—H15A	0.9600
C3—C4	1.390 (3)	C15—H15B	0.9600
C3—H3A	0.9300	C15—H15C	0.9600
			
C4—O1—C15	117.86 (19)	O2—C8—N2	120.23 (18)
C10—O3—H3	109.5	O2—C8—C9	121.91 (18)
C12—O4—H4	109.5	N2—C8—C9	117.85 (16)
C7—N1—N2	114.96 (16)	C14—C9—C10	117.00 (17)
C8—N2—N1	120.15 (16)	C14—C9—C8	117.74 (17)
C8—N2—H2A	120 (2)	C10—C9—C8	125.23 (17)
N1—N2—H2A	120 (2)	O3—C10—C11	119.10 (16)
C6—C1—C2	118.3 (2)	O3—C10—C9	120.06 (16)
C6—C1—C7	119.57 (19)	C11—C10—C9	120.82 (17)
C2—C1—C7	122.1 (2)	C12—C11—C10	120.26 (17)
C3—C2—C1	120.2 (2)	C12—C11—H11	119.9
C3—C2—H2	119.9	C10—C11—H11	119.9
C1—C2—H2	119.9	O4—C12—C11	122.14 (18)
C2—C3—C4	121.0 (2)	O4—C12—C13	117.50 (18)
C2—C3—H3A	119.5	C11—C12—C13	120.36 (18)
C4—C3—H3A	119.5	C14—C13—C12	118.52 (19)
O1—C4—C5	125.0 (2)	C14—C13—H13	120.7
O1—C4—C3	115.5 (2)	C12—C13—H13	120.7
C5—C4—C3	119.5 (2)	C13—C14—C9	122.94 (18)
C4—C5—C6	119.1 (2)	C13—C14—H14	118.5
C4—C5—H5	120.4	C9—C14—H14	118.5
C6—C5—H5	120.4	O1—C15—H15A	109.5
C1—C6—C5	121.9 (2)	O1—C15—H15B	109.5
C1—C6—H6	119.0	H15A—C15—H15B	109.5
C5—C6—H6	119.0	O1—C15—H15C	109.5
N1—C7—C1	123.69 (19)	H15A—C15—H15C	109.5
N1—C7—H7	118.2	H15B—C15—H15C	109.5
C1—C7—H7	118.2		

### Hydrogen-bond geometry (Å, °)

**Table:**

*D*—H···*A*	*D*—H	H···*A*	*D*···*A*	*D*—H···*A*
N2—H2A···O3	0.901 (10)	1.94 (2)	2.646 (2)	134 (3)
O4—H4···O2^i^	0.82	1.85	2.671 (2)	174
O3—H3···N1^ii^	0.82	2.48	3.234 (2)	154
O3—H3···O2^ii^	0.82	2.14	2.788 (2)	136

Symmetry codes: (i) *x*+1/2, −*y*+7/2, *z*; (ii) *x*+1/2, −*y*+5/2, *z*.
